# Genome and Transcriptome Sequencing of *casper* and *roy* Zebrafish Mutants Provides Novel Genetic Clues for Iridophore Loss

**DOI:** 10.3390/ijms21072385

**Published:** 2020-03-30

**Authors:** Chao Bian, Weiting Chen, Zhiqiang Ruan, Zhe Hu, Yu Huang, Yunyun Lv, Tengfei Xu, Jia Li, Qiong Shi, Wei Ge

**Affiliations:** 1 Centre of Reproduction, Development and Aging, Faculty of Health Sciences, University of Macau, Taipa, Macau 999078, China; bianchao@genomics.cn (C.B.); 201701014@jyu.edu.cn (W.C.); yb47610@connect.um.edu.mo (Z.H.); 2 Shenzhen Key Lab of Marine Genomics, Guangdong Provincial Key Lab of Molecular Breeding in Marine Economic Animals, BGI Academy of Marine Sciences, BGI Marine, BGI, Shenzhen 518083, China; ruanzhiqiang@genomics.cn (Z.R.); huangyu@genomics.cn (Y.H.); lvyunyun@njtc.edu.cn (Y.L.); xutengfei1@genomics.cn (T.X.); li081766@gmail.com (J.L.); 3 School of Life Sciences, Jiaying University, Meizhou 514015, China; 4 BGI Education Center, University of Chinese Academy of Sciences, Shenzhen 518083, China

**Keywords:** *casper*, *roy*, genome sequencing, transcriptome sequencing, variation, iridophore degeneration

## Abstract

*casper* has been a widely used transparent mutant of zebrafish. It possesses a combined loss of reflective iridophores and light-absorbing melanophores, which gives rise to its almost transparent trunk throughout larval and adult stages. Nevertheless, genomic causal mutations of this transparent phenotype are poorly defined. To identify the potential genetic basis of this fascinating morphological phenotype, we constructed genome maps by performing genome sequencing of 28 zebrafish individuals including wild-type AB strain, *roy orbison* (*roy*), and *casper* mutants. A total of 4.3 million high-quality and high-confidence homozygous single nucleotide polymorphisms (SNPs) were detected in the present study. We also identified a 6.0-Mb linkage disequilibrium block specifically in both *roy* and *casper* that was composed of 39 functional genes, of which the *mpv17* gene was potentially involved in the regulation of iridophore formation and maintenance. This is the first report of high-confidence genomic mutations in the *mpv17* gene of *roy* and *casper* that potentially leads to defective splicing as one major molecular clue for the iridophore loss. Additionally, comparative transcriptomic analyses of skin tissues from the AB, *roy* and *casper* groups revealed detailed transcriptional changes of several core genes that may be involved in melanophore and iridophore degeneration. In summary, our updated genome and transcriptome sequencing of the *casper* and *roy* mutants provides novel genetic clues for the iridophore loss. These new genomic variation maps will offer a solid genetic basis for expanding the zebrafish mutant database and in-depth investigation into pigmentation of animals.

## 1. Introduction

Laboratory zebrafish has been one of the most widely utilized models for developmental biology, genetics, in vivo imaging, and modeling of human genetic diseases, owing to its rapid development, high fecundity, and juvenile transparency [1,2,3,4]. However, the wild-type zebrafish strains, such as AB and Tübingen, have opaque trunks in their adult stage. This limits their usage in some research such as in vivo imaging of cancer engraftment. To resolve the problem of this deficiency, a transparent *casper* mutant that lacks both light-absorbing melanophores and reflective iridophores was generated by crossing *nacre* and *roy orbison* (*roy*) mutants [5]. The *nacre* is a white mutant that carries a mutation in the gene of *mitfa* (microphthalmia-associated transcription factor) [6], leading to a recessive phenotype of a complete loss of black melanophores. However, how this inactivated *mitfa* regulates or impacts its upstream and downstream genes is still largely unknown.

The *roy* mutant shows a recessive phenotype with complete loss of iridophores and partial reduction of melanophores. Recent studies using cDNA cloning reported that *mpv17* (a gene encoding a mitochondrial inner membrane protein) in *casper* and *roy* mutants had a 19-bp deletion in its mRNA (between exons 2 and 3), which is possibly responsible for the iridophore loss [7,8]. A *mpv17*-knockdown zebrafish exhibited a moderate loss of iridophores along the body axis [8]. A recent study [9] further demonstrated that the *mpv17*^−/−^ zebrafish knockouts exhibited iridophore loss. It was therefore postulated that the *mpv17* gene in zebrafish could play a key role in pyrimidine synthesis for iridophore development and maintenance [9]. Its detailed genome lesions have not been sequenced either. It is thus interesting to identify and characterize the precise genomic mutations in the *mpv17*, as well as to check transcriptional changes of genes upstream and downstream of the *mpv17* for understanding of the genetic mechanisms of iridophore loss.

**Figure 1:**
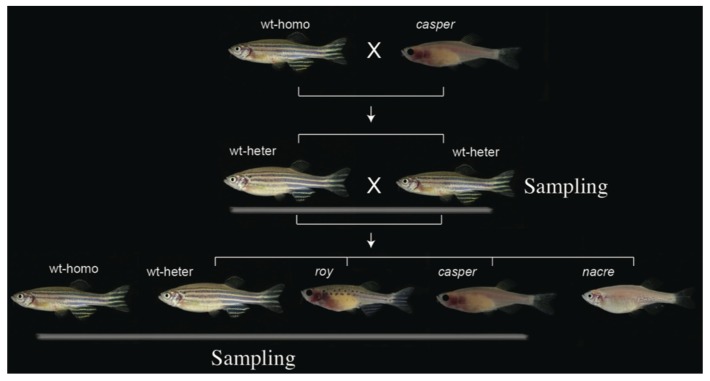
Construction of the *casper* pedigree. Four groups, including *casper*, *roy*, wt-heter, and wt-homo (as the outgroup), were sampled for our present study.

Moreover, homozygous mutations in zebrafish can often yield diverse phenotype defects in various tissues, some of which are potentially related to human diseases [10,11]. Previous studies have reported massive genetic mutation maps for strains of Tübingen, WIK, AB, and TLF [12,13,14,15,16]. However, the genomic resources for the *roy* and *casper* strains are still limited. In this study, we first performed genome sequencing of 24 individuals, including ten *casper*, ten *roy*, and four wild-type heterozygotes (wt-heter) from a pedigree crossed between *casper* and wild-type zebrafish (see Figure 1). We also sequenced four wild-type homozygotes of AB zebrafish (wt-homo; no relation to the above pedigree). Lastly, novel high-resolution variation maps for both *casper* and *roy* mutants were constructed. These maps contained approximately 4.3 million (M) of homozygous single nucleotide polymorphisms (SNPs) and 1.1 M of insertions and deletions compared to the wild-type Tübingen strain reference genome (Zv9) [17]. Among these homozygous SNPs, we identified genomic mutations in the *mpv17* introns within the linkage-disequilibrium (LD) block of chromosome (chr) 20. We also demonstrated that there are many novel non-synonymous SNPs that result in residue changes or even inactivation of target genes. These mutations are potentially involved in some morphological changes, like abnormal vision responses [18], which have not previously been investigated in the *casper* and *roy* mutants. Therefore, these genetic resources are very valuable in molecular guidance and interpretation for discovery of new morphological changes. This may also facilitate more practical applications of the *casper* and *roy* mutants.

## 2. Results

### 2.1. Summary of the Genome Sequencing Data

To maximally reduce individual and group variations that were not related to the transparent phenotype, a *casper* pedigree was constructed (Figure 1) for this study. Ten *casper*, ten *roy*, and four wt-heter were sequenced. Four wt-homo individuals that had no relationship with the individuals from the aforementioned pedigree were also sequenced as the outgroup. Approximately 661.7 Gb of raw reads from the 28 samples were generated (Table 1). After a series of filtering steps, 452.7 Gb of clean reads were mapped onto the reference genome (Zv9) of the Tübingen strain [17]. On average, the mapping ratio across the 28 individuals was about 85.0%; around a ten-fold mapping depth was observed, and the reference genome coverage was 91.5% (Table S1).

A total of 4.3 M of high-quality homozygous SNPs were identified across the four groups (*casper*, *roy*, wt-homo, and wt-heter; Figure S2). Among them, 4.0 M were determined to be novel when compared to the public Ensembl database, which contained 1.3 M of zebrafish SNPs [19,20]. SNP density was calculated to be approximately one SNP per 0.3 kb (Figure S1). This was an intermediate value near to the lowest compared to previous reports of one SNP within 0.2~41 kb [12,13,14,15,16]. Because we sequenced the AB strain and used it as the reference for genome assembly, SNPs from the wt-homo group could be considered as variants from the AB and Tübingen strains.

Subsequent variant annotations demonstrated that 2.3 M of SNPs were localized within intergenic regions, 1.9 M of SNPs were present in intron regions, and only 79,879 (with 55,573 synonymous and 24,306 non-synonymous SNPs) appeared in exonic regions. A total of 1.1 M of homozygous Indels were also identified (see detailed distributions in Figure S3). These variants are potentially important for the identification of morphological divergence that has not yet been investigated.

**Figure 2:**
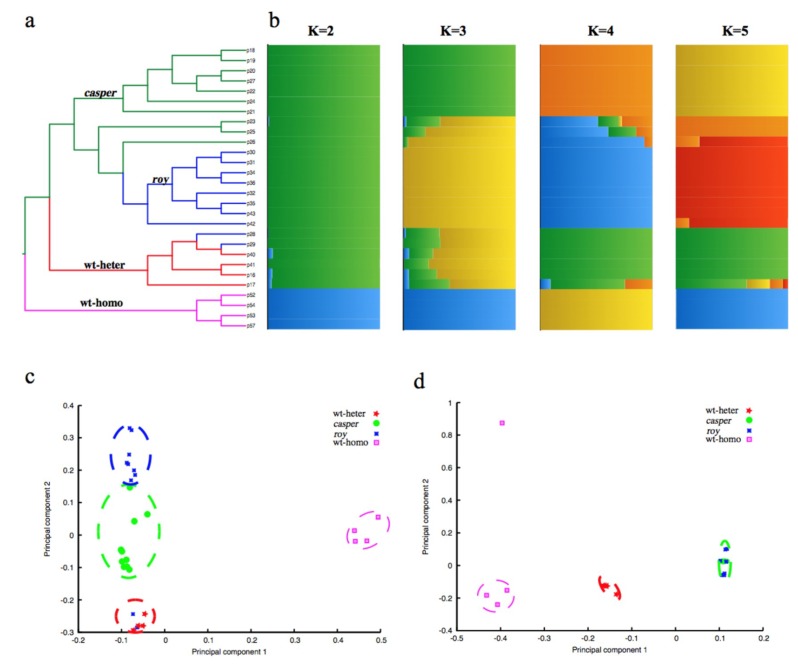
Pedigree structure of the 28 individuals. (**a**) A neighbor-joining phylogenetic tree of the 28 individuals. The branches with green, blue, red, and purple colors represent the *casper*, *roy*, wt-heter, and wt-homo groups, respectively. (**b**) Pedigree structure of the 28 individuals. The x-axis shows the subgroup membership, and the y-axis exhibits the corresponding individuals from the phylogenetic tree. (**c**) Principal component analysis (PCA) of the 28 individuals, using the entire homozygous SNPs. Four groups were identified as follows: *casper* in green, *roy* in blue, wt-heter in red, and wt-homo in purple. (**d**) PCA of the 28 individuals using homozygous SNPs from the linkage disequilibrium (LD) block in chr20. It appears that all the individuals of both *casper* and *roy* strains were merged into one group.

Further neighbor-joining tree construction (Figure 2a) and principal component analysis (PCA; Figure 2c) confirmed clustering of these 28 individuals into four groups on the basis of those identified homozygous SNPs. Structural analysis for admixture estimation with the selected parameter of *K* = 2 (Figure 2b) revealed two stable groups comprising a group of 24 individuals from the pedigree of *casper*, *roy* and wt-heter, and the other group of the four wt-homo individuals. At *K* = 4, all the 28 individuals were clearly divided into four groups. Results of these population analyses were consistent with our sampling strategy (see Figure 1), and confirmed that the identified SNPs indeed reflected the divergence among the four sequenced groups. These SNPs can therefore be used for molecular investigations in the identification of molecular mechanisms leading to the transparent phenotype.

### 2.2. Genome-Wide Association Study (GWAS) and Transcriptome Results for Melanin Loss

**Figure 3:**
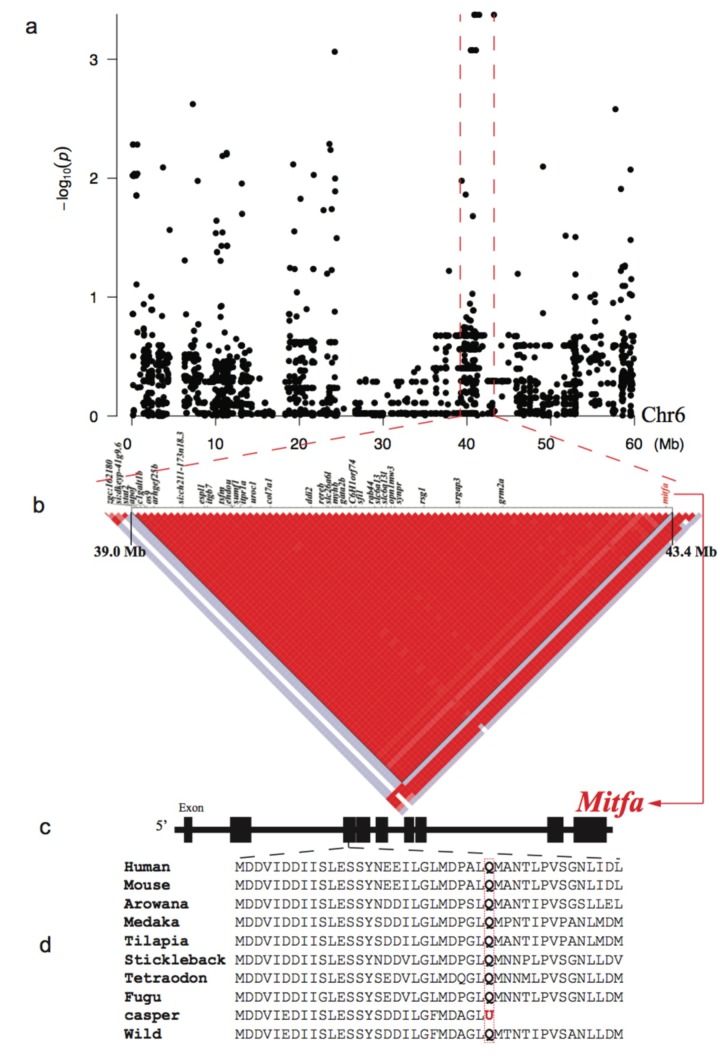
Genome-wide association study (GWAS) and LD results for the melanin loss. (**a**) A Manhattan plot of the GWAS data. The x- and y-axes represent localization in the chr6 and *p*-values, respectively. The red dashed lines marked the LD block in chr6. (**b**) A Haplotype block with complete linkage disequilibrium (LD, *r*^2^ = 1) and its component genes within the chr6. Red and white spots indicate strong (*r*^2^ = 1) and weak (*r*^2^ = 0) LDs, respectively. (**c**) Detailed structure of the *mitfa* gene. Black boxes represent the exons. (**d**) A nonsense mutation (g. chr6:43360485 C>T; p. Q112U) in the *mitfa* of the *casper* strain. Note that the wt-homo and all other vertebrates have a glutamine at this site.

SNPs within the 39.0-43.4 Mb region of chr6 were found to be significantly associated (*p* < 10^−3^) with the melanin loss phenotype (Figure 3a). Interestingly, a linkage analysis of this region revealed a special single haplotype block with complete linkage disequilibrium (*r*^2^ = 1; Figure 3b).

In total, 5484 private SNPs were identified in the *casper* group. Among them, 85 were non-synonymous SNPs located in the exons of 41 genes within the identified LD block (Table S2). Among these SNPs, only one private SNP in the *casper* group was found to be a nonsense mutation (g. chr6:43360485 C>T) that led to a premature termination codon (p. Q112U) in exon 3 of the *mitfa* gene. Analysis of the skin transcriptome identified 241 up-regulated and 337 down-regulated genes in the *casper* individuals compared with the wt-homo counterparts (Table S7). The down-regulated genes were significantly enriched (*p* ≤ 0.05) in four ‘Gene Ontology’ (GO) terms, which included melanosome organization (GO:0032438; Figure 4 and Table S9). In addition, we established a *casper* specific haplotype and non-synonymous SNP set near the *mitfa* gene in the LD block of chr6 (Table S2), which will facilitate further investigations on this special mutant.

### 2.3. GWAS and Transcriptome Results for Iridophore Loss

The *roy* strain has a spontaneous mutation that leads to complete iridophore defects and a transparent trunk [5,8,9]. As both *casper* and *roy* mutants have the same phenotype of iridophore loss, they were treated as the same transparent group. Unlike the nonsense SNP identified within the *mitfa* gene of the *casper* mutant, no specific homozygous nonsense SNPs were discovered in both *casper* and *roy* mutants. GWAS analysis with a recessive model was performed on homozygous SNPs between the transparent group and the other two groups. SNPs within a 33.1–39.1 Mb region of chr20 were confirmed to have the most significant association (*p* < 10^−5^) with the iridophore loss phenotype (Figure 5a).

In this region, our linkage analysis demonstrated a special single haplotype block with complete LD (*r*^2^ = 1; Figure 5b) that specifically appeared in both *casper* and *roy* groups. Conversely, the wt-homo individuals did not have this haplotype, and all the wt-heter individuals were heterozygous for this haplotype. A total of 83 private missense SNPs were identified in 39 genes within this LD block from the *roy* and *casper* groups (Figure 5b, Table S3). The PCA result, analyzed using entire homozygous SNPs from this LD block, also showed that both *casper* and *roy* individuals were clustered into one group (Figure 2d). It seems that this region might be causal for the phenotype of iridophore loss.

In total, 1065 specific homozygous Indels were identified in this LD block for the *casper* and *roy* groups. No Indels, however, were located within exonic regions. Interestingly, the *mpv17* gene was also identified in this block and it carried 46 specific homozygous SNPs in its intronic regions in both *casper* and *roy* groups (Table S4). All these specific SNPs showed major allele frequency at 1.0 in the both groups, independently. Conversely, the wt-homo group did not have any of these mutations, while its half alleles carried these mutations. The *mpv17* gene in the *capser* and *roy* groups also contained two specific homozygous insertions (g. chr20: 38737392 T>TA and g. chr20: 38738626 C>CT; Figure S4b,d) and one specific homozygous deletion (g. chr20:38737515 T>-; Figure S4c) within intron 5. These insertions and deletions have been confirmed by Sanger sequencing (Figure S4 and Table S5).

The specific 19-bp deletion in the *mpv17* mRNA of both *casper* and *roy* groups was also confirmed in our present study (Figure S4a), which is consistent with previous reports [7,8]. Interestingly, transcription levels of the *mpv17* in both *casper* and *roy* groups were remarkably lower compared to the wt-homo group (decrease over three-fold; Table S8). A detailed distribution of the transcript reads mapped on the *mpv17* gene clearly demonstrated translational frameshifts in this gene of both *casper* and *roy* groups (Figure 6b,c). Several other genes (such as *paics*, *ednrb1a*, *gart*, *gmps,* and *ltk*) potentially associated with the iridophore loss were also examined [7,8,21,22,23,24]. However, no specific mutation was detected in these genes of the *casper* and *roy* groups. Lastly, 68 up-regulated and 178 down-regulated genes were detected in the transcriptome data from both *casper* and *roy* groups compared to the wt-homo group (Table S8). The down-regulated genes were significantly enriched (*p* ≤ 0.05) in three GO terms, including “cellular amino acid metabolic process” (GO:0006520), “cellular modified amino acid catabolic process” (GO:0042219), and “heparan sulfate proteoglycan biosynthetic process and enzymatic modification” (GO:0015015; Table S10).

## 3. Discussion

### 3.1. Transcriptome Regulation and Evolution of mitfa Involved in the Melanophore Loss

Wild-type zebrafish (such as AB and Tübingen strains) have three basic types of pigment cells [25], including reflective iridophores, yellow xanthophores, and black melanophores [26,27]. The *casper* mutant, inheriting both recessive phenotypes of the *roy* and *nacre* mutants, lost iridophores and melanophores. In the present study, we found that only one private SNP of the *casper* group was a nonsense mutation (g. chr6:43360485 C>T), which led to a premature termination codon (p. Q112U) in the exon 3 of the *mitfa* gene (Figure 3d). This confirms a previous report [6], but we provide more comprehensive genomic and transcriptome details for the involvement of the *mitfa* gene in the *nacre* mutant, one of the two mutations that lead to full transparency of the *casper* zebrafish. It is well documented that the *mitfa* gene is a “master regulator” of melanocyte development and plays a key role in neural crest cell fate specification [28,29,30]. Therefore, the nonsense mutation detected in this study is expected to result in functional inactivation of the *mitfa*, subsequently leading to a defect in melanin synthesis.

**Figure 4:**
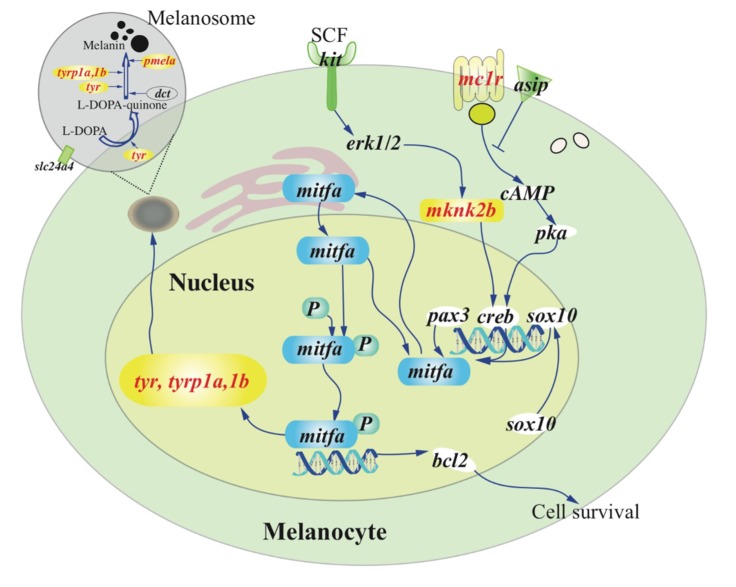
A speculated view of the melanin synthesis pathway. Genes marked in red were down-regulated in the *casper* compared with the wt-homo group. The non-functional *mitfa* suppresses the expression of *tyr*, *tyrp1a*, and *1b* genes, which in turn lead directly to melanophore death in the *casper*. Detailed transcription levels (FPKM values) of these related genes were listed in Table S11.

As a regulatory factor, the *mitfa* gene consistently maintained a very low level of transcription in all genotypes examined, including the wt-homo, *roy* and *casper* groups (Table S11). As expected, the important genes that work downstream of the *mitfa*, such as *tyr* (tyrosinase), *tyrp1a* and *tyrp1b* (tyrosinase-related protein 1a and 1b), and *pmela* (premelanosome protein), all showed low or even no transcription in the *casper* group. The transcription of *tyr* and *tyrp1b* genes was undetectable (FPKM = 0), and *tyrp1a* showed a much lower value than the wt-homo group. These genes encode key enzymes for melanin synthesis [31] and have been well documented to be transcriptional targets of *mitfa* [30]. Moreover, a recent study [32] reported that a missense mutation (Arg->Cys) in *tyrp1a* caused melanophore death in zebrafish. We thus propose that melanin synthesis in the *casper* zebrafish is non-functional due to the nonsense mutation and functional inactivation of *mitfa*, which reduces expression of *tyr*, *tyrp1a* and *tyrp1b*, ultimately leading to melanophore death in the *casper* mutant (Figure 4). Interestingly, several other genes that work upstream of *mitfa* in melanin synthesis pathway including *mknk2b* (MAP kinase-interacting serine/threonine-protein kinase 2b) and *mc1r* (melanocortin 1 receptor), showed remarkably lower transcription levels in the *casper* group compared with those genes in the wt-homo group (Figure 4 and Table S11).

In contrast to mammals including humans and mice that have only one *mitf* gene in their genomes [33], two isotypes of the *mitf* genes (named *mitfa* and *mitfb*) are present in majority of teleost genomes [17]. A phylogenetic tree of *mitfa* and *mitfb* from 20 representative species (Figure S5) clearly clustered teleosts into one main branch that was diverged from mammals, leading to formation of two independent branches. These data suggest that both *mitf* genes could be paralogs generated from teleost-specific genome duplication (TSGD) event(s) [34,35]. The *mitfa* of *casper* has a nonsense mutation that leads to the loss of melanocytes; in contrast, the *mitfb* gene is involved in the development of retinal pigment in zebrafish [36], while no private variant in its coding regions was identified in the present study, suggesting that *mitfb* could not compensate for the functional loss of *mitfa*. In mice, mutations in the *mitf* gene can induce various defects in melanocytes, retinal pigment epithelium and mast cells, and osteoclasts [37,38,39]. We therefore hypothesize that the pair of duplicated *mitf* genes generated by the TSGD underwent subfunctionalization with two paralogous *mitf* genes to take up independent functions. Both genes have subsequently been preserved in various teleost genomes.

### 3.2. A Possible Genetic Basis for the Iridophore Loss

**Figure 5:**
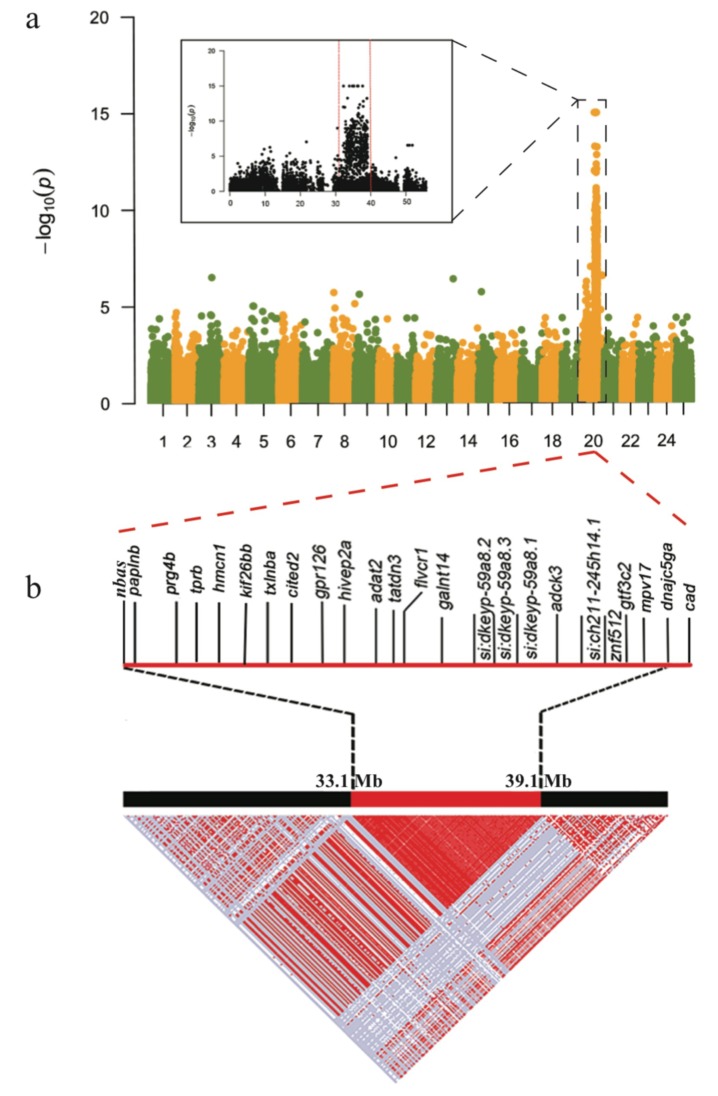
GWAS and LD results for the iridophore degeneration. (**a**) A Manhattan plot of the GWAS data. The x- and y-axes represent localization of all chromosomes and *p*-values, respectively. (**b**) A Haplotype block with complete linkage disequilibrium (LD, *r*^2^ = 1) and its component genes within chr20. Red and white spots represent strong (*r*^2^ = 1) and weak (*r*^2^ = 0) LDs, respectively.

**Figure 6:**
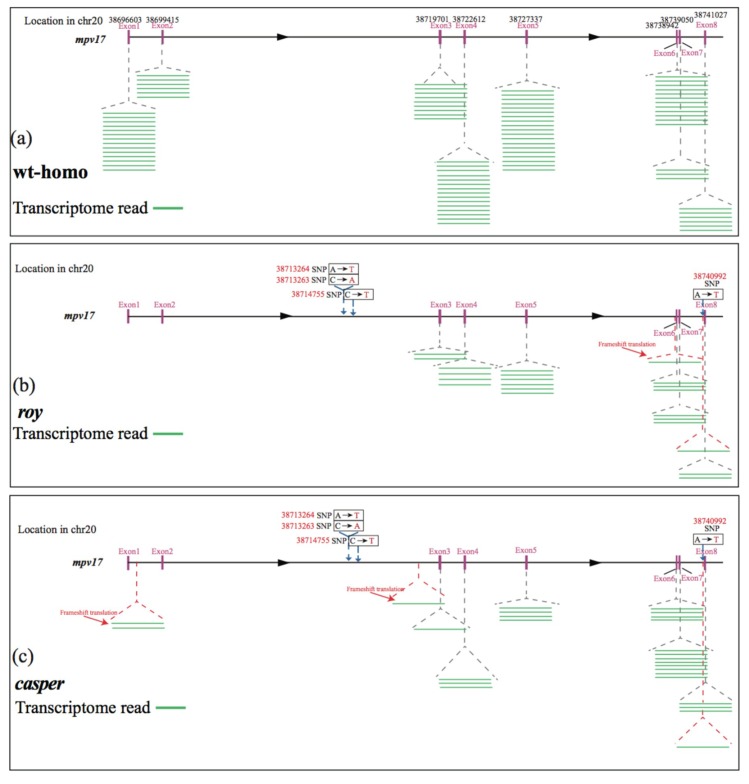
Deletions in the *mpv17* gene. Note the distribution differences of transcriptome reads in the *mpv17* gene from the wild-type (**a**), *roy* (**b**), and *casper* (**c**) strains. Green lines represent the transcriptome reads, while red arrows highlight the frameshifted reads.

Both *casper* and *roy* mutants lack reflective iridophores, rendering them the see-through phenotype [5]. Mainly containing guanine to act as reflective platelets, iridophores usually generate many crystals that are located in iridosomes [27]. In the present study, we identified a specific single haplotype block with complete LD in both *casper* and *roy* (Figure 5b), which was tightly associated with the iridophore loss. It was reported that the loss of iridophores in both *casper* and *roy* might be associated with a 19-bp deletion in their *mpv17* mRNA [8]. However, this deletion was not proven at the genomic level. Interestingly, we could not find such a deletion in the genomes of both *casper* and *roy*; however, we discovered that the *mpv17* gene in both groups harbors 46 specific homozygous SNPs in the intronic regions of this LD block (Table S4). It was speculated in a previous study that a deletion could be present within the intron 2 of *mpv17* in *casper* and *roy*. This deletion was suggested to lead to a 19-bp deletion between exons 2 and 3 in the *mpv17* mRNA [8]. This speculation is not supported by our present study, because only three SNPs were present in the intron 2 of the *mpv17* gene (Figure 6b, c). According to the models recently reviewed by Anna et al. [40], intronic mutations may affect pre-mRNA splicing, these SNPs within the intron 2 of the *mpv17* gene in *casper* and *roy* might cause for exon fragment skipping (mis-splicing), resulting in a 19-bp deletion in the mature mRNA.

Interestingly, our transcriptome data showed that the transcription levels of the *mpv17* in the *casper* and *roy* groups were remarkably lower than the wt-homo group (decrease over three-fold; Table S8). In addition, our data also demonstrated several frameshifts in the *mpv17* transcripts (Figure 5b,c). Similarly, purine nucleoside phosphorylase 4a (*pnp4a*) in the purine metabolism pathway, a marker for iridophore development that can metabolize guanosine to guanine [24], also showed lower transcription in both *casper* and *roy* than the wt-homo group. Accumulation of *pnp4a* transcripts can increase transcription of genes for guanosine metabolism in zebrafish iridophores, while knockdown of *pnp4a* generated loss of iridophores in the trunk and eyes [23]. The inactivation of *mpv17* could weaken deoxyribonucleotide metabolism and impair dNTP availability [9]. This may reduce materials and energy for the purine metabolism pathway, mainly via the suppressed expression of the *pnp4a* gene, and finally leading to loss of reflective pigments and degeneration of iridophores.

## 4. Materials and Methods

### 4.1. Sample Preparation and Sequencing

A *casper* pedigree was constructed (Figure 1) by crossing *casper* and AB strains (wild-type homozygote, wt-homo). Two F1 offspring (a female and a male), wild-type heterozygotes (wt-heter) with the recessive alleles for *roy* and *nacre*, were then crossbred to yield the F2 generation. The F2 offspring showed trait segregations to result in four various strains, including *casper*, *roy*, *nacre,* and wild-type (homozygotes and heterozygotes). They were kept in flow-through aquaria with a ZebTEC system (Tecniplast, Buguggiate, Italy) at 28 ± 1 °C under a 14L:10D lighting cycle. All fishes were fed twice daily with commercial fish food by a robotic feeding Tritone (Tecniplast, Buguggiate, Italy).

Genomic DNA was extracted from muscle tissue of 28 individuals (ten *casper*, ten *roy*, four wt-heter, and four wt-homo) using Qiagen genomicsTip100 (Qiagen, Gaithersburg, MD, USA) in accordance with the manufacturer’s instructions. We employed the routine whole-genome shotgun sequencing strategy and constructed 350-bp libraries of the 28 samples. Paired-end sequencing (150 bp in length) was subsequently performed on an Illumina HiSeq X-Ten platform (Illumina, San Diego, CA, USA). All experiments were endorsed by the Research Ethics Committee of University of Macau and BGI-Shenzhen (approval ID: FT18134). Approximately 650.7 Gb of raw reads were generated. After trimming off 15 bases at both ends of the reads and removing reads with gaps of 10 bp or more, a total of 452.7 Gb of clean data (Table S1) were obtained for further analysis.

### 4.2. Read Alignment and Variant Calling

The clean reads were aligned onto the zebrafish reference genome (Zv9) using the Burrows- Wheeler Aligner (BWA, version 0.6.2) [41] with the following optimized parameters: aln -n 0.04 -o 1 -e 30 -i 15 -d 10 -l 35 -k 2 -m 2000000 -t 4 -M 3 -O 11 -E 4 -R 30 -q 0 -I. SAMtools (version 1.2) [42] was employed to sort each alignment and filter out PCR duplicates. Subsequently, the Genome Analysis Toolkit (GATK, version 2.8-1) [43] was utilized to process indel realignments for improvement of the next SNP and indel calling step. The GATK with a parameter of “-T UnifiedGenotyper” was applied for SNP calling (Figures S1 and S2) and a parameter of “-glm INDEL” for indel calling (Figure S3) in all the 28 individuals.

To obtain a more accurate SNP set, we performed a series of filtering steps to remove many reads with the following thresholds: a haplotype score >13.0, Fisher strand bias >60.0, read depth of SNP >2.5-fold and <40.0-fold (1/4- and 4-fold of the average depth), miss ratio of the 28 datasets <30.0%, and intervals of >5 bp between each two SNPs. Homozygous SNPs were counted with their major allele frequency at 1.0 in each independent group. These identified homozygous SNP sets from the four sequenced groups were further annotated as SNPs in 5′ UTRs, 3′ UTRs, intergenic sequences, and intronic and exonic regions based on their locations in the zebrafish reference genome. Moreover, exonic region SNPs were further divided into synonymous, nonsynonymous SNPs (leading to amino acid changes; Tables S2 and S3), and nonsense SNPs (resulting in premature stop codons).

### 4.3. Phylogenetic, Population Structure, and Principal Component Analyses

The homozygous SNPs were selected as the input data for subsequent phylogenetic, population structure and principal component (PCA) analyses. To construct the phylogenetic tree across these 28 samples, we employed PLINK (version 1.07) [44] to calculate the matrix of pairwise genetic distances and utilized a neighbor-joining method to build the phylogenetic tree. FigTree (version 1.4.2, http://tree.bio.ed.ac.uk/software/figtree/) was applied to draw the final phylogenetic tree (Figure 2a).

The genetic structure was illustrated using Frappe (version 1.1) [45] with algorithms constructed for expectation maximization and block relaxation, and analyses with 10,000 interactions. The K parameters were adapted and ranged from 2 to 5 for the investigation of potential genetic relationships among the 28 individuals (Figure 2b). Simultaneously, the PCA analysis was performed by EIGENSOFT (version 3.0) [46], and the remarkable factors of eigenvectors were calculated using the Tracey–Widom test in R package (Figure 2c,d).

### 4.4. Transcriptome Sequencing and mRNA Quantification

Total RNA was extracted from muscle and skin tissues of two wt-homo, two *roy,* and two *casper* individuals using TRIzol reagent (Invitrogen, Carlsbad, CA, USA). Transcriptome sequencing (RNA-Seq) libraries of these six samples were prepared using a TruSeq RNA sample preparation kit (Illumina). All independent libraries were sequenced on an Illumina HiSeq 2500 platform to generate 125-bp paired-end reads. Subsequently, total mRNA reads of each sample were aligned onto the zebrafish reference genome (Zv9) by using the Tophat software (version 2.0.9) [47]. FPKM (Fragments Per Kilobase of exon per Million fragments mapped) values were calculated using Cufflink (version 2.0.2. Linux_x86_64) [48] for mRNA quantification. All detailed transcription values were listed in Table S6.

Differentially expressed genes (DEGs) between *casper* and wt-homo or between transparent groups (*capser* and *roy*) and wt-homo samples were examined by the edgeR software [49]. Fold changes >2 were set as the threshold for the DEG identification (Tables S7 and S8). Gene Ontology (GO) enrichment analysis of up- and down-regulated genes was performed using GOrilla [50] with *p* ≤ 0.001 (Table S9).

### 4.5. GWAS Analysis and LD Blocks Identification

For the genome-wide association study (GWAS), EMMAX [51] with the mixed linear model (MLM) was employed to detect associations among the homozygous SNPs. Due to the fact that only *casper* has the melanin loss phenotype, the GWAS analysis with the recessive model for melanin loss phenotype was performed on homozygous SNPs between *casper* and the other three integrated groups. As both *casper* and *roy* mutants have the same phenotype of iridophore loss, we merged them into a transparent group for the GWAS analysis of iridophore loss phenotype. Score assignment of phenotypic traits of each group in the GWAS analysis included: 1 for wt-homo and wt-heter and *roy* and 2 for *casper* in the phenotype of melanin loss; and 1 for wt-homo and wt-heter and 2 for *casper* and *roy* in the phenotype of iridophore loss.

Significance levels of genotype-phenotype association (*p*) were calculated using the Fisher’s exact test under a recessive model. The kinship of each population was examined by the function of emmax-kin with default parameters. The input file of Q matrix from above population structures was calculated by Frappe [45]. Subsequently, the most notable SNPs from the GWAS analysis were sent for linkage disequilibrium (LD) analysis. Haploview [52] was employed to calculate the LD correlation coefficient (r^2^) values for drawing LD block plots (Figure 3b and Figure 5b).

## 5. Conclusions

Using the whole-genome sequencing approach, we generated a genetic variant map for 28 zebrafish individuals from *casper*, *roy*, wt-homo, and wt-heter groups. Applying a series of rigorous bioinformatics methods to the genetic data generated in this study, we confirmed some results from previous studies. However, we provided more in-depth details for both *casper* and *roy* groups at a genomic level, including identification of specific genomic SNPs in *mpv17* introns, high confidence determination of novel mutations and LD blocks, and potential genetic effects of these findings. Additionally, transcriptome sequencing provided detailed evidence for changes in transcription of both downstream and upstream genes influenced by the inactivated *mitfa* and *mpv17* genes. In summary, our genome and transcriptome sequencing of both *casper* and *roy* zebrafish mutants provide novel genetic insights into the iridophore loss. Our genomic, transcriptomic, and variant data will definitely expand the catalog of genetic resources for zebrafish strains so as to promote research on teleost biology and human diseases.

## Figures and Tables

**Table 1:** Summary of genetic variations in the four sequenced groups.

Parameter	*casper*	*roy*	wt-homo (AB)	wt-heter
Individual number	10	10	4	4
Raw bases (Gb)	217.68	248.01	91.13	104.91
Clean bases (Gb)	159.03	167.59	62.2	63.86
Homozygous SNPs (Mb)	1.60	1.83	2.66	2.55
Synonymous SNPs (bp)	26,212	28,745	32,334	33,147
Non-synonymous SNPs (bp)	11,583	12,832	14,376	14,515
Private homozygous SNPs * (bp)	157,920	165,901	1,375,693	438,220
Homozygous Indels	350,678	407,000	630,190	665,766
Private homozygous Indels *	32,193	35,018	285,790	112,184

* Private homozygous single nucleotide polymorphisms (SNPs) and Indels are defined as those homozygous SNPs and Indels that were only identified in one group.

## Supplementary Materials

Supplementary materials can be found online at https://www.mdpi.com/1422-0067/21/7/2385/s1. Figure S1. Density distributions of homozygous SNPs from the casper, roy, wt-heter and wt-homo groups. Figure S2. A Circos view of the distributions of homozygous SNPs within the four sequenced groups. Figure S3. A Circos view of the distributions of homozygous Indels. Figure S4. Validation of insertions and deletions in the mRNA and introns of mpv17 gene by PCR amplification and Sanger sequencing. Figure S5. A phylogenetic tree of mitfa and mitfb from 20 representative vertebrates. Table S1. Summary of map ratios and mapped reads of the 28 sequenced individuals. Table S2. Detailed descriptions of the nonsynonymous SNPs in the LD region of chr6. Table S3. Detailed descriptions of the nonsynonymous SNPs in the LD region of chr20. Table S4. Detailed descriptions of the SNPs in the mpv17 gene. Table S5. Primer pairs for validation of indels by Sanger sequencing. Table S6. Transcription value (FPKM) of each gene in the skin tissues of the casper, roy and wt-homo groups, respectively. Table S7. Up-regulated and down-regulated genes and their corresponding transcription values (FPKM) in the casper compared with the wt-homo group. Table S8. Up-regulated and down-regulated genes and their corresponding transcription values (FPKM) in the casper & roy groups compared with the wt-homo group. Table S9. GO enrichment of down-regulated genes in the casper compared to the wt-homo group. Table S10. GO enrichment result of down-regulated genes of casper and roy groups compared to wt-homo group. Table S11. Detailed transcription values (FPKM) of down-regulated genes related to melanin synthesis in the casper compared to the wt-homo group (red for down-regulated genes). Accession number for the transcriptome (RNA-seq) and genome (DNA-seq) data reported in this paper is PRJNA427078 in National Center for Biotechnology Information (NCBI).

## Abbreviations

chr	chromosome
*ednrb1a*	endothelin-receptor b1a
*gart*	glycinamide ribonucleotide transformylase
*ltk*	leucocyte tyrosine kinase
*gmps*	guanine monophosphate synthase
GWAS	genome-wide association study
LD	linkage-disequilibrium
M	million
*mc1r*	melanocortin 1 receptor
*mitf*	microphthalmia-associated transcription factor
*mknk2b*	MAPK interacting serine/threonine kinase 2b
*mpv17*	mitochondrial inner membrane protein MPV17
*paics*	phosphoribosylaminoimidazole carboxylase
PCA	principal component analysis
*pmela*	premelanosome protein
*pnp4a*	purine nucleoside phosphorylase 4a
SNP	single nucleotide polymorphism
TSGD	teleost-specific genome duplication
*tyr*	tyrosinase
*tyr1*	tyrosinase-related protein 1
wt-heter	wild-type heterozygote
