# Supplementary material for: Genome and Transcriptome Sequencing of casper and roy Zebrafish Mutants Provides Novel Genetic Clues for Iridophore Loss
Source: Int J Mol Sci. 2020 Mar 30;21(7):2385. doi: 10.3390/ijms21072385 (PMC7177266; doi:10.3390/ijms21072385)
Supplement: Supplementary file 1 [file ijms-21-02385-s001.zip › Supplementary Information.pdf]

Supplementary Information

Supplementary Figures

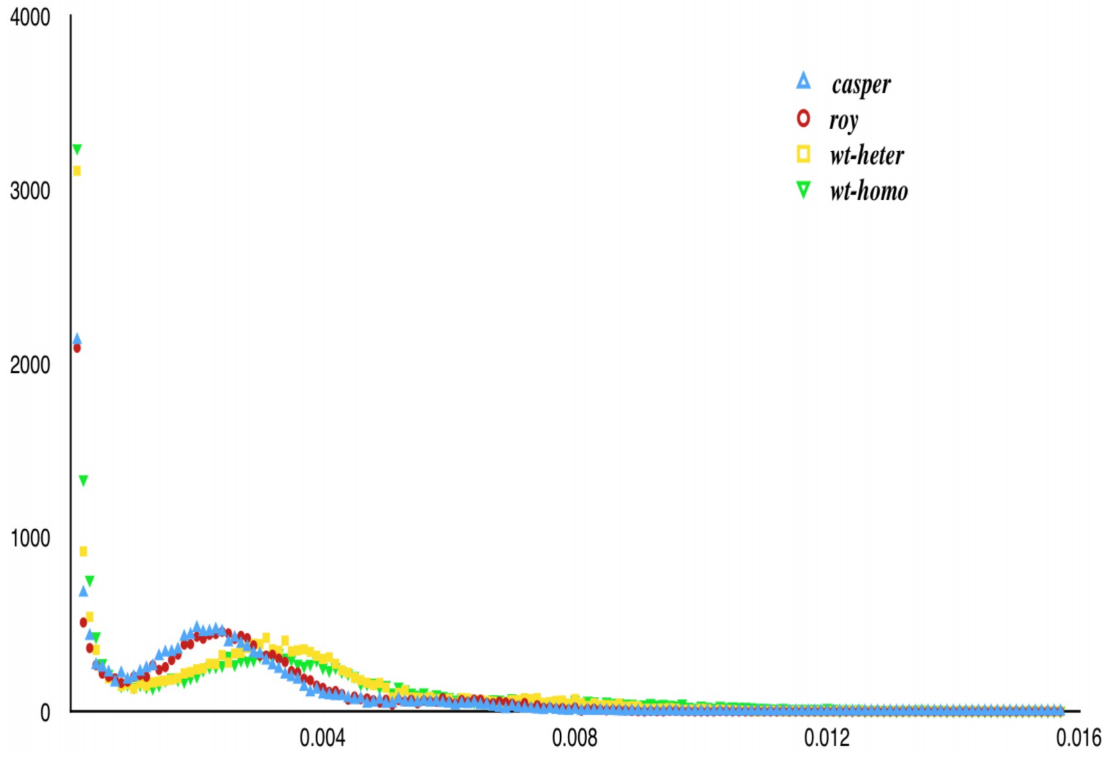

Figure S1. Density distributions of homozygous SNPs from the *casper*, *roy*, wt-heter and wt-homo groups. The density was calculated in Non-overlapping 50-kb windows. The x axis represents homozygous SNP density, and the y axis represents window numbers.

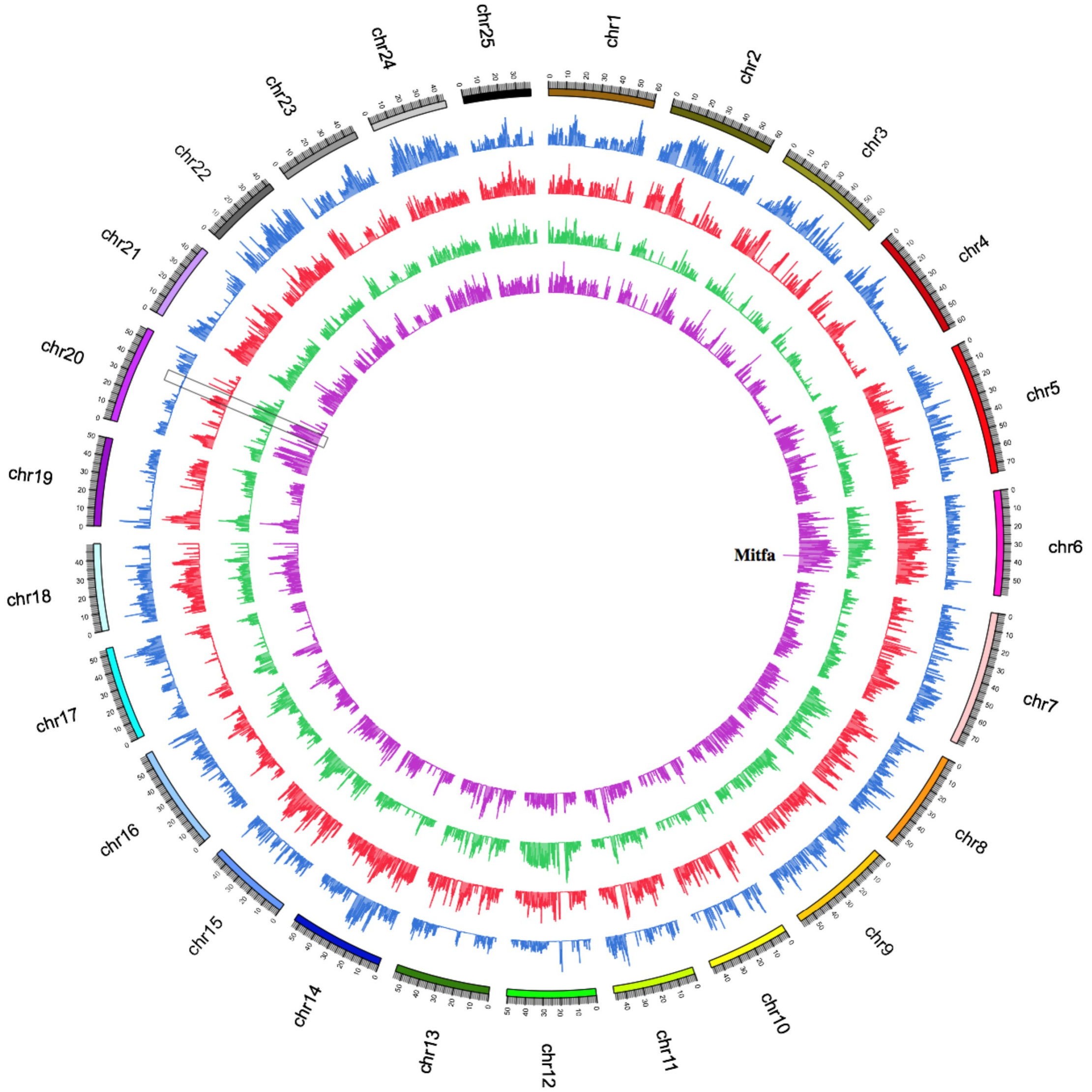

Figure S2. A Circos view of the distributions of homozygous SNPs within the four sequenced groups. The blue, red, green and purple circles represent the distribution of SNPs in the wt-homo, wt-heter, *roy* and *casper*, respectively. The black box stands for the LD region in the chr20. Note the *mitfa* gene, which was localized in the chr6.

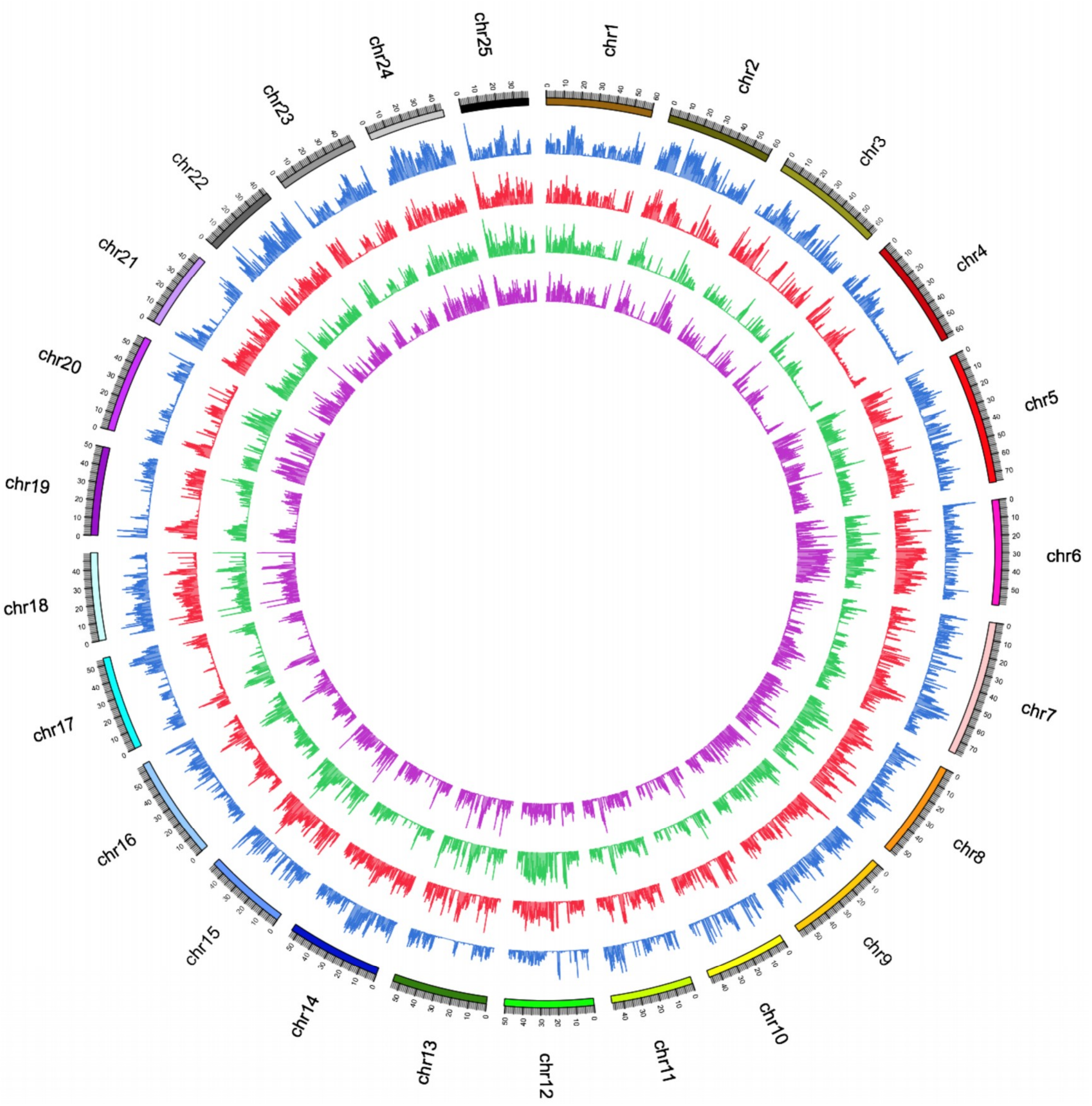

Figure S3. A Circos view of the distributions of homozygous Indels. The blue, red, green and purple circles represent the distribution of Indels in the wt-homo, wt-heter, *roy* and *casper*, respectively.

a. The reported 19-bp deletion in the *mpv17* mRNA.

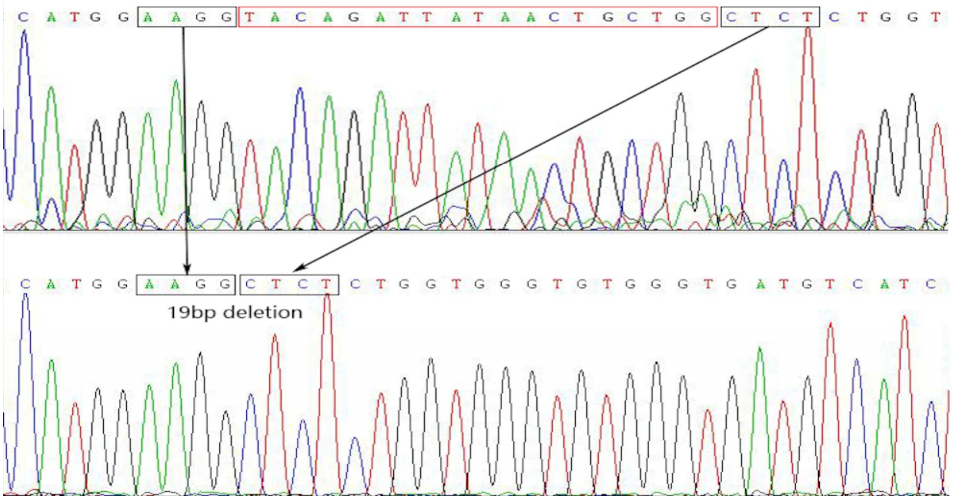

b. chr20: 38737392 T > TA

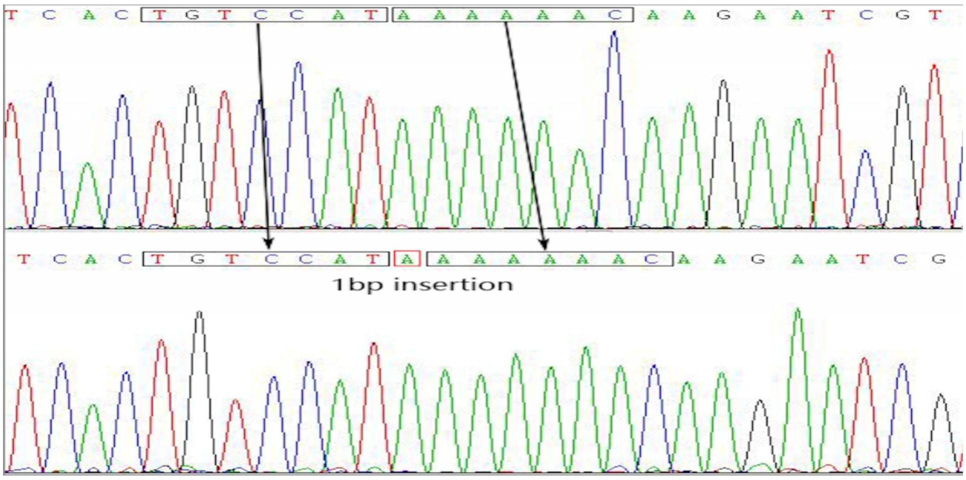

c. chr20: 38737515 T > -

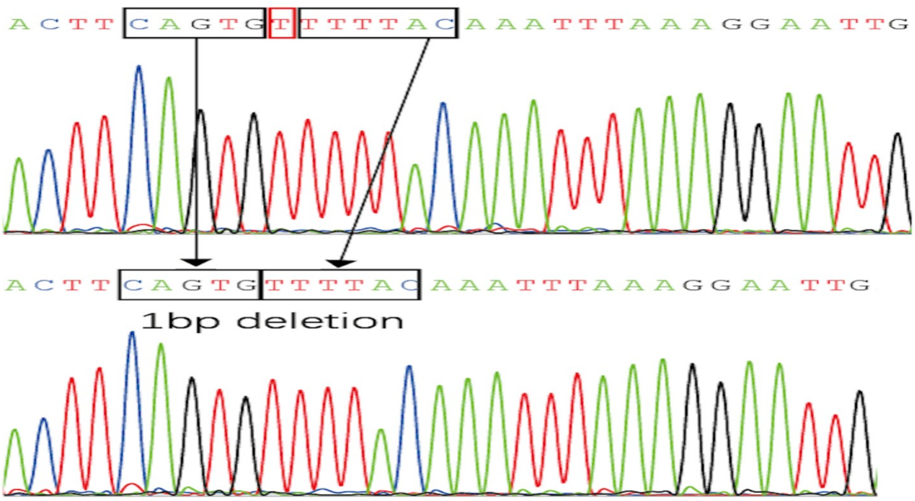

d. chr20: 38738626 C > CT

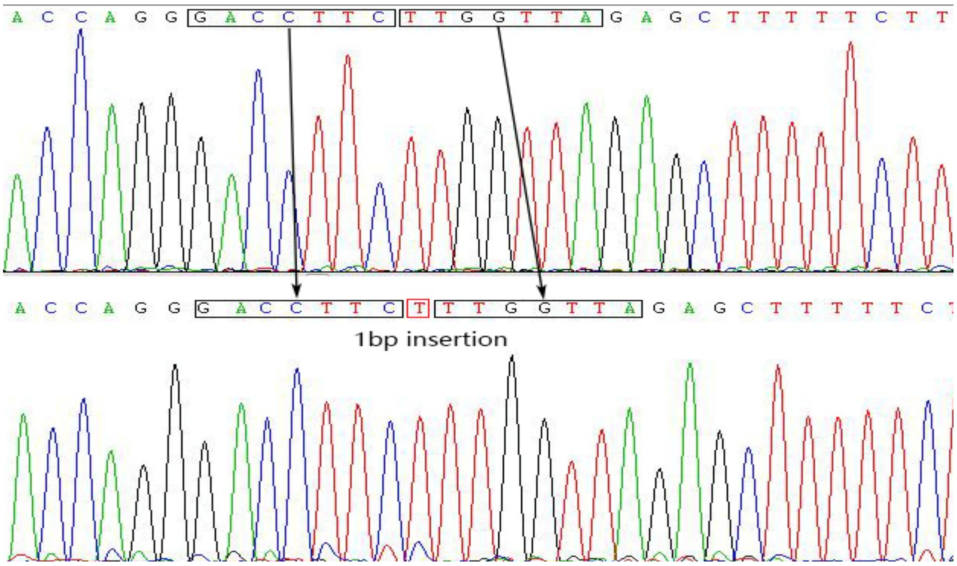

Figure S4. Validation of insertions and deletions in the mRNA and introns of *mpv17* gene by PCR amplification and Sanger sequencing. Detailed primer pairs were provided in **Table S5**.

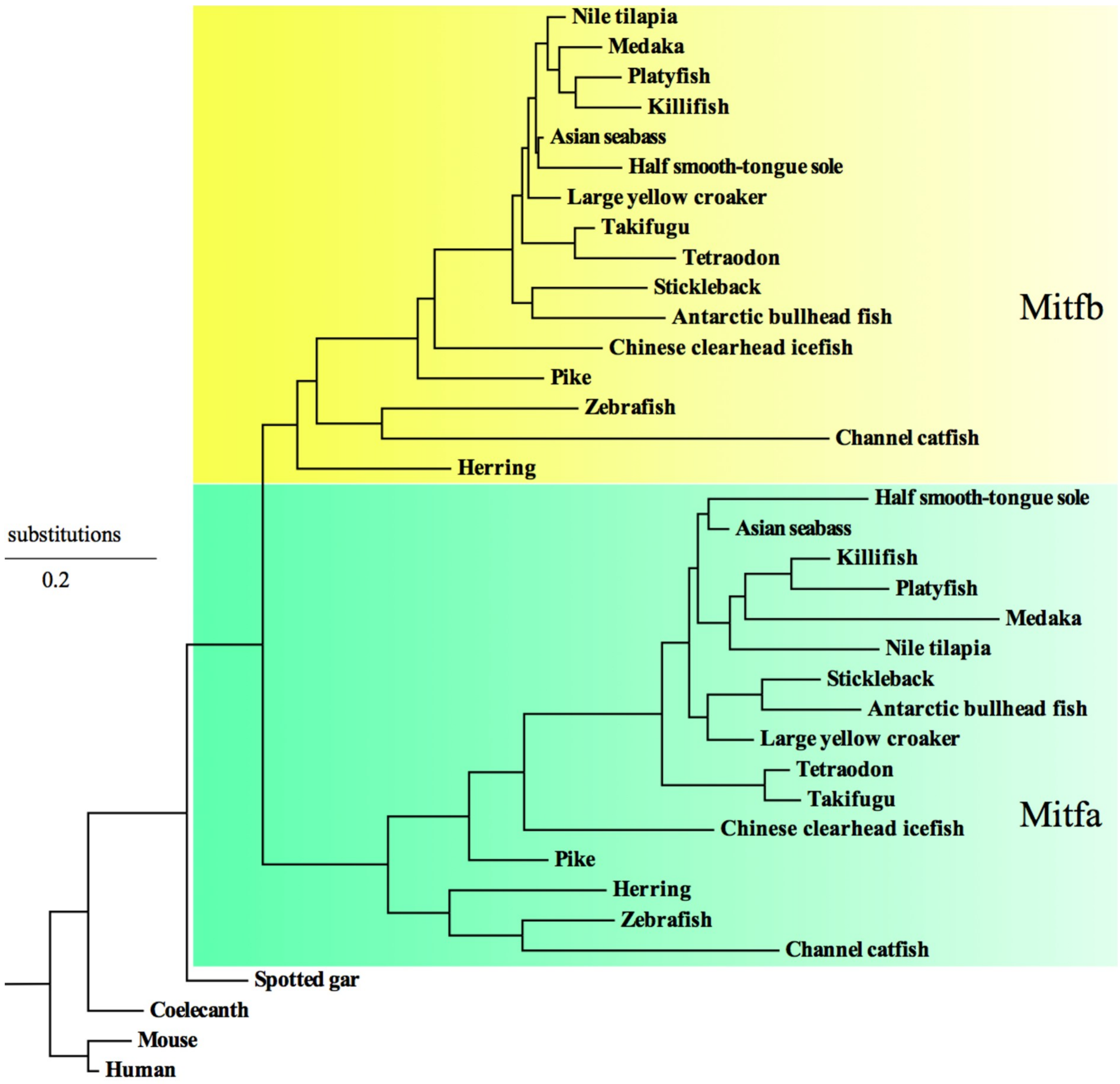

Figure S5. A phylogenetic tree of Mitfa and Mitfb from 20 representative vertebrates. These examined animals include Mouse, Human, Coelecanth, Spotted gar, Channel catfish, Zebrafish, Herring, Pike, Chinese clearhead icefish, Takifugu, Tetraodon, Large yellow croaker, Antarctic bullhead fish, Stickleback, Nile tilapia, Medaka, Platyfish, Killifish, Asian seabass, and Half smooth-tongue sole (data were downloaded from the Ensemble 87). Mitfa and Mitfb protein sequences were aligned by MUSCLE (version 3.8.31; Edgar 2004). The PhyML (version 3.1; Criscuolo 2011) and FigTree (version 1.4.3; http://tree.bio.ed.ac.uk/software/figtree) were used to construct the phylogenetic tree.

Supplementary Tables

Table S1. Summary of map ratios and mapped reads of the 28 sequenced individuals.

Library	Sample	Mapped reads	Map ratio	Coverage	Depth (×)
wHAXPI045366-52	wt-homo♂1	108,011,424	85.60%	91.94	9.50
wHAXPI045368-53	wt-homo♂2	108,694,940	85.72%	92.42	9.56
wHAXPI045369-54	wt-homo♀3	108,922,427	85.37%	91.33	9.57
wHAXPI045370-57	wt-homo♀4	117,802,419	85.52%	91.42	10.37
wHAXPI032631-16	wt-heter♂1	132,865,266	85.86%	92.80	11.65
wHAXPI032632-17	wt-heter♀2	104,368,516	85.41%	92.20	9.14
wHAXPI033188-22	wt-heter♂3	111,402,892	84.60%	92.48	9.75
wHAXPI033189-23	wt-heter♀4	104,529,733	84.59%	92.20	9.16
wHAXPI032633-18	*casper ♂1*	116,942,037	85.19%	90.94	10.23
wHAXPI032634-19	*casper♂2*	116,528,495	84.63%	90.28	10.17
wHAXPI032635-20	*casper♂3*	121,643,546	84.44%	90.36	10.60
wHAXPI032636-21	*casper♂4*	130,655,210	85.66%	91.39	11.43
wHAXPI032637-22	*casper♂5*	126,388,094	86.39%	92.58	11.08
wHAXPI032638-23	*casper♀6*	105,309,814	83.72%	89.94	9.15
wHAXPI032639-24	*casper♀7*	142,158,979	85.50%	92.54	12.47
wHAXPI032640-25	*casper♀8*	123,011,882	85.61%	92.63	10.78
wHAXPI032641-26	*casper♀9*	103,239,335	83.63%	88.03	9.00
wHAXPI032642-27	*casper♀10*	101,138,546	84.61%	89.03	8.84
wHAXPI032643-28	*roy♂1*	105,585,269	85.46%	90.89	9.24
wHAXPI032644-29	*roy♂2*	102,509,665	85.64%	92.07	8.98
wHAXPI032645-30	*roy♂3*	104,569,119	85.47%	91.79	9.16
wHAXPI032646-31	*roy♀4*	98,224,617	84.89%	90.78	8.60
wHAXPI032647-32	*roy♀5*	136,149,429	85.00%	92.77	11.94
wHAXPI032648-34	*roy♀6*	110,458,807	85.11%	92.50	9.67
wHAXPI032649-35	*roy♀7*	110,634,267	83.34%	87.21	9.63
wHAXPI032650-36	*roy♀8*	106,561,342	85.31%	91.96	9.33
wHAXPI033190-24	*roy♂9*	125,558,613	83.95%	93.11	10.99
wHAXPI033191-25	*roy♂10*	124,435,837	84.75%	93.14	10.88

wt-homo: wild type homozygote; wt-heter: wild type heterozygote;

♂: male individual; ♀: female individual.

Table S2. Detailed descriptions of the nonsynonymous SNPs in the LD region of chr6.

(*See the separate Excel workbook*)

Table S3. Detailed descriptions of the nonsynonymous SNPs in the LD region of chr20.

(*See the separate Excel workbook*)

Table S4. Detailed descriptions of the SNPs in the *mpv17* gene.

(*See the separate Excel workbook*)

Table S5. Primer pairs for validation of indels by Sanger sequencing.

Mutation	Forward Primer	Reverse Primer
mRNA 19-bp deletion	AGGCTCTGATGGCCAAACACCCAT	GAGGTAAGAGTTCCAGACAACAGC
g. chr20: 38737392 T > TA	TACATAAAATGTCCTTTAGAATGA	TTTGGGTTTATTTTAGAAAGGTTA
g. chr20: 38738626 C > TC	ATGCTGCATTTGAGTAACGGTTGG	TTGTGAAATGGAAAAAGCATAGTA
g. chr20: 38737515 T > -	AACATCCTGTCAGACATATTCAGA	ATTGCTTCAGGCACAAAAGTAAAT

Table S6. Transcription value (FPKM) of each gene in the skin tissues of the *casper*, *roy* and wt-homo groups, respectively.

(*See the separate Excel workbook*)

Table S7. Up-regulated and down-regulated genes and their corresponding transcription values (FPKM) in the *casper* compared with the wt-homo group.

(*See the separate Excel workbook*)

Table S8. Up-regulated and down-regulated genes and their corresponding transcription values (FPKM) in the *casper & roy* groups compared with the wt-homo group.

(*See the separate Excel workbook*)

Table S9. GO enrichment of down-regulated genes in the *casper* compared to the wt-homo group.

GO term	Description	*p*-value	Gene Symbol
GO:0042558	pteridine-containing compound metabolic process	2.00E-5	*gch2*, *zgc:153031*, *qdpra*, *aldh1l2*
GO:0032438	melanosome organization	1.56E-4	*tyrp1b, tyrp1a, pmela*
GO:0006520	cellular amino acid metabolic process	4.12E-4	*aspg, bcat1, ccbl1, farsa, aldh18a1, qdpra, pcyox1, arg2, phgdh, asns*
GO:0044281	small molecule metabolic process	6.25E-4	*bcat1, nudt15, rrm2b, plcd3a, pnp4a, ugp2a, arg2, mat2ab, gch2, ccbl1, aldh18a1, phgdh, asns, aldh1l2, urah, aspg, farsa, zgc:153031, sprb, cnp, slc7a3a, qdpra, pcyox1*

Table S10. GO enrichment result of down-regulated genes of *casper* and *roy* groups compared to wt-homo group.

GO term	Description	pvalue	Gene Symbol
GO:0006520	cellular amino acid metabolic process	3.93E-5	*aspz, bcat1, farsa, aldh18a1, pcyox1, arg2, phgdh,*
GO:0042219	cellular modified amino acid catabolic process	1.86E-4	*pcyox1, chac1, aldh1l2*
GO:0015015	heparan sulfate proteoglycan biosynthetic process, enzymatic modification	4.4E-4	*hs6st3b, hs2st1a*

Table S11. Detailed transcription values (FPKM) of down-regulated genes related to melanin synthesis in the *casper* compared to the wt-homo group (red for down-regulated genes).

Gene ID	*casper1*	*casper2*	*roy1*	*roy2*	wt-homo1	wt-homo2	Gene Symbol
ENSDARG00000003732	0.53	0.80	1.07	1.09	0.52	0.65	*mitfa*
ENSDARG00000039077	0	0	0.35	0.06	0.17	0.44	*tyr*
ENSDARG00000029204	0.10	0.18	1.25	0.54	1.04	1.13	*tyrp1a*
ENSDARG00000056151	0	0	3.84	3.64	4.07	4.38	*tyrp1b*
ENSDARG00000020237	0.19	0.17	0.81	0.32	1.59	1.06	*mc1r*
ENSDARG00000018411	1.55	1.69	1.71	2.24	2.29	2.03	*mknk1*
ENSDARG00000011373	0.92	0.57	0.72	0.59	0.82	1.18	*mknk2a*
ENSDARG00000015164	3.82	4.15	2.50	0.66	15.93	44.10	*mknk2b*
ENSDARG00000091298	0.13	0.07	2.71	2.06	1.76	2.64	*pmela*
ENSDARG00000077467	4.54	4.37	4.08	3.48	3.39	4.24	*sox10*
ENSDARG00000015425	0	0	0.06	0	0	0	*slc24a4a*
ENSDARG00000067509	0.06	0	0.11	0	0.37	0.10	*slc24a4b*
ENSDARG00000056133	0	0	0.04	0	0.03	0	*kita*
ENSDARG00000043317	0.08	0.29	0.13	0.20	0.16	0.13	*kitb*
ENSDARG00000027552	0.73	0.90	0.62	0.31	1.11	4.40	*erk2*
ENSDARG00000070573	12.97	16.85	12.03	10.70	13.26	14.99	*erk1*
ENSDARG00000053586	5.62	5.82	6.23	6.55	4.82	3.96	*creb*
ENSDARG00000077858	1.15	0.49	1.01	3.18	2.03	1.99	*asip*
ENSDARG00000060596	2.54	3.66	2.62	1.22	2.62	3.38	*pka1*
ENSDARG00000060641	0	0	0.16	0.11	0	0	*pka2*
ENSDARG00000025613	0.38	0	0	0.17	0	0	*bcl2*
ENSDARG00000010192	0	0	0	0	0	0.05	*pax3a*
ENSDARG00000028348	0.46	0.05	0.17	0	0	0	*pax3b*

Reference

Criscuolo A (2011) morePhyML: improving the phylogenetic tree space exploration with PhyML 3. *Mol Phylogenet Evol* **61**, 944-948.

Edgar RC (2004) MUSCLE: multiple sequence alignment with high accuracy and high throughput. *Nucleic Acids Res* **32**, 1792-1797.
